# Supplementary figures and images for: Human presence and infrastructure impact wildlife nocturnality differently across an assemblage of mammalian species
Source: PLoS One. 2023 May 25;18(5):e0286131. doi: 10.1371/journal.pone.0286131 (PMC10212153; doi:10.1371/journal.pone.0286131)

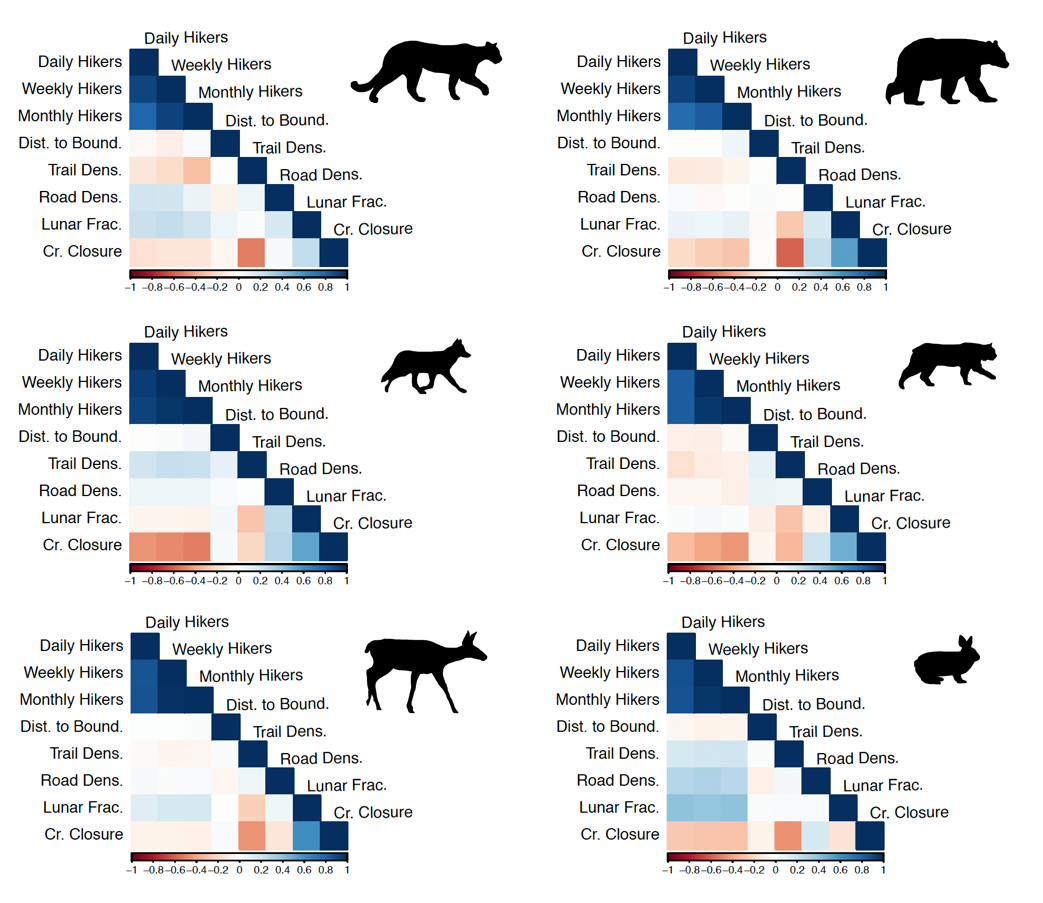

Supplement: S1 Fig — Species included cougars (top left), black bears (top right), coyotes (middle left), bobcats (middle right), black-tailed deer (bottom left), and snowshoe hares (bottom right). For all species, daily human detections, and weekly and monthly human detection rates were highly collinear (all R > |0.7|; min. = 0.76). Maximum correlation values aside from human detection variables varied by species, but all were < |0.7| (max. cougar R = -0.51, crown closure and trail density; max. black bear R = -0.58, crown closure and trail density; max. black-tailed deer R = 0.61, crown closure and lunar fraction, max. snowshoe hare R = -0.45, crown closure and trail density; max. coyote R = 0.53, crown closure and lunar fraction; and max. bobcat R = 0.48, crown closure and lunar fraction). (PNG) [file pone.0286131.s001.png]

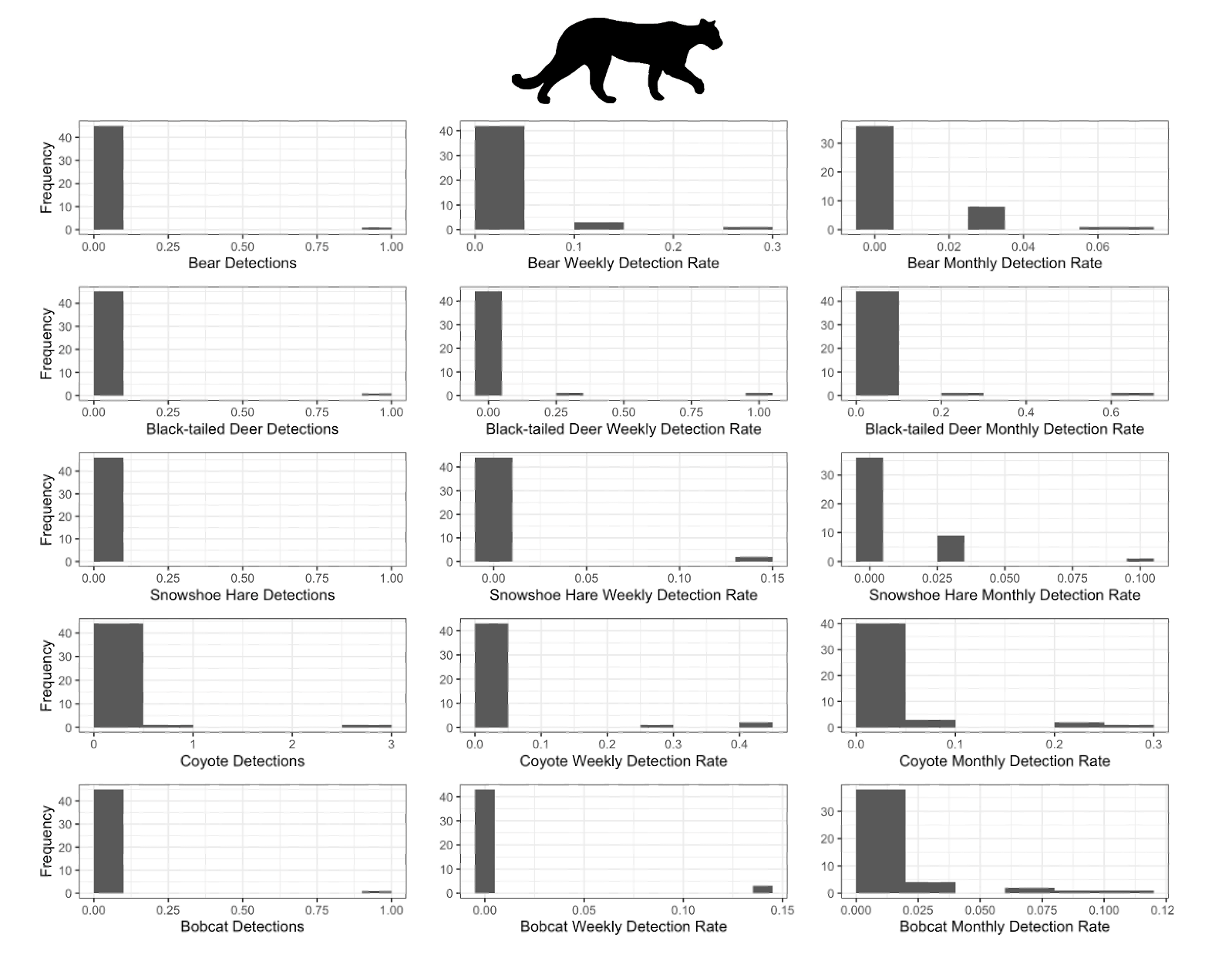

Supplement: S2 Fig — The cougar dataset comprises all independent detection events of cougars. Therefore, histograms show the detections of each species during that same day of the detection event (left column), the weekly detection rate during that same week of the detection event (middle column), and the monthly detection rate during that same month of the detection event (right column). Non-cougar species are black bears, black-tailed deer, snowshoe hares, coyotes, and bobcats (rows, from top to bottom). (PNG) [file pone.0286131.s002.png]

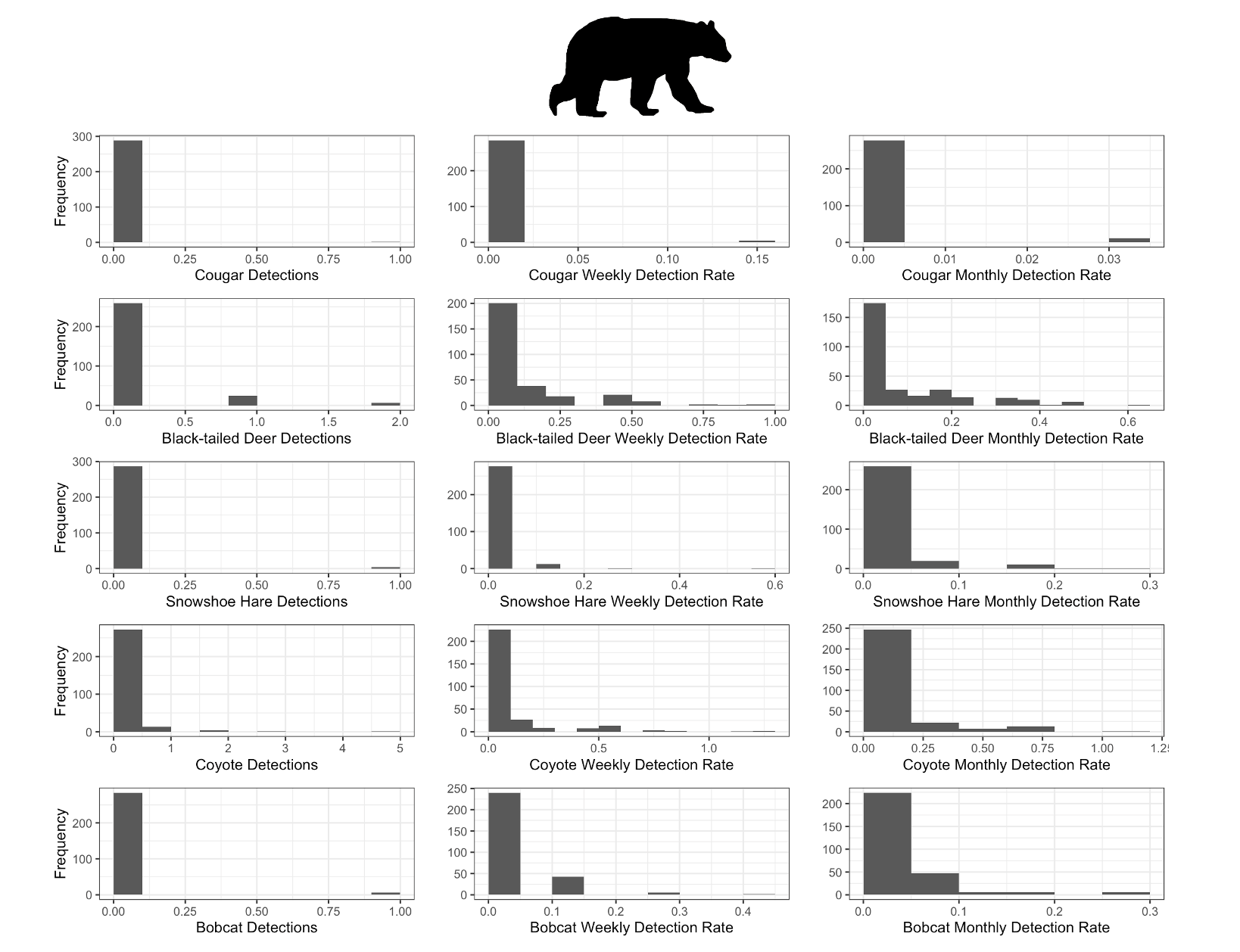

Supplement: S3 Fig — The black bear dataset comprises all independent detection events of black bears. Therefore, histograms show the detections of each species during that same day of the detection event (left column), the weekly detection rate during that same week of the detection event (middle column), and the monthly detection rate during that same month of the detection event (right column). Non-black bear species are cougars, black-tailed deer, snowshoe hares, coyotes, and bobcats (rows, from top to bottom). (PNG) [file pone.0286131.s003.png]

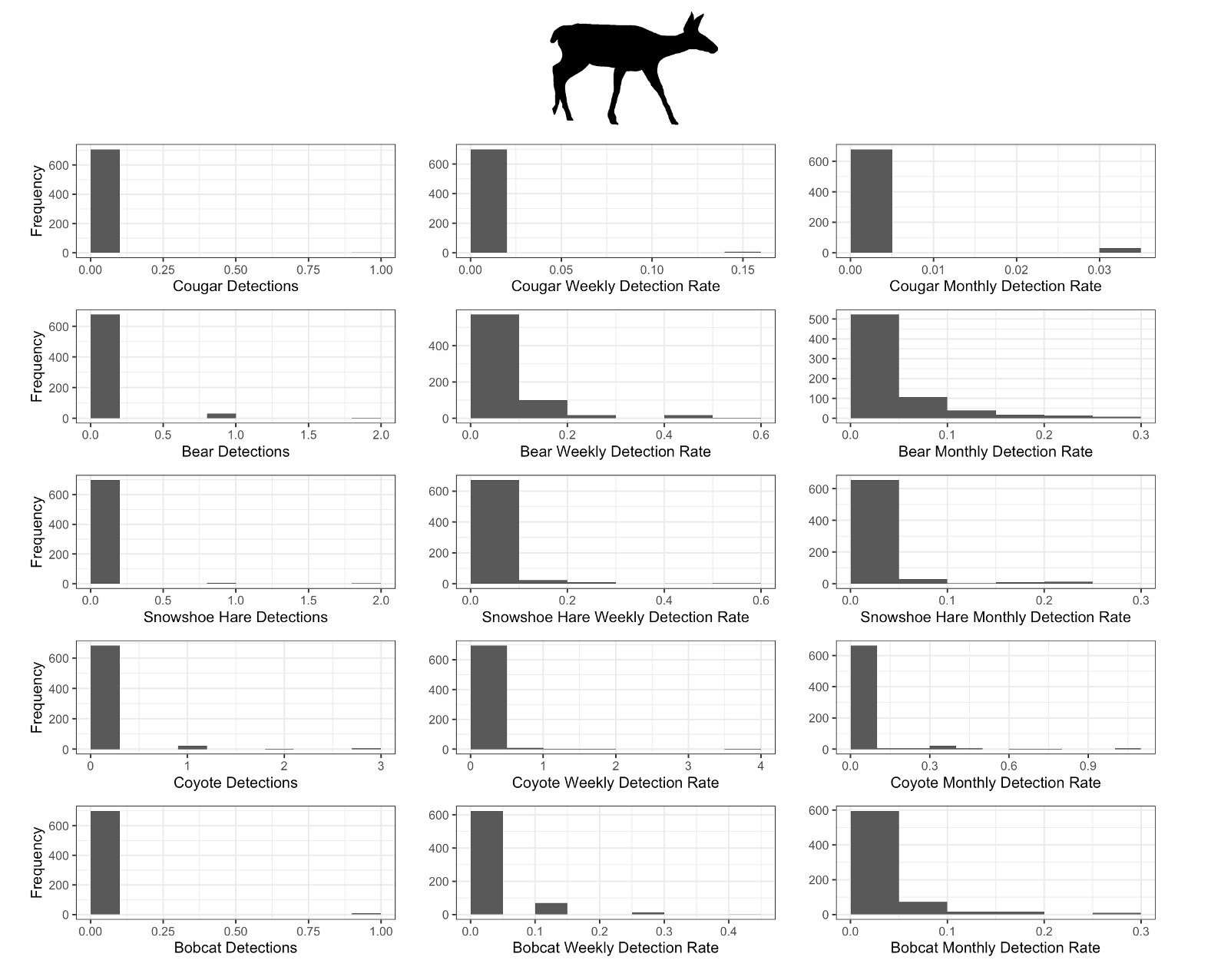

Supplement: S4 Fig — The black-tailed deer dataset comprises all independent detection events of black-tailed deer. Therefore, histograms show the detections of each species during that same day of the detection event (left column), the weekly detection rate during that same week of the detection event (middle column), and the monthly detection rate during that same month of the detection event (right column). Non-black-tailed deer species are cougars, black bears, snowshoe hares, coyotes, and bobcats (rows, from top to bottom). (PNG) [file pone.0286131.s004.png]

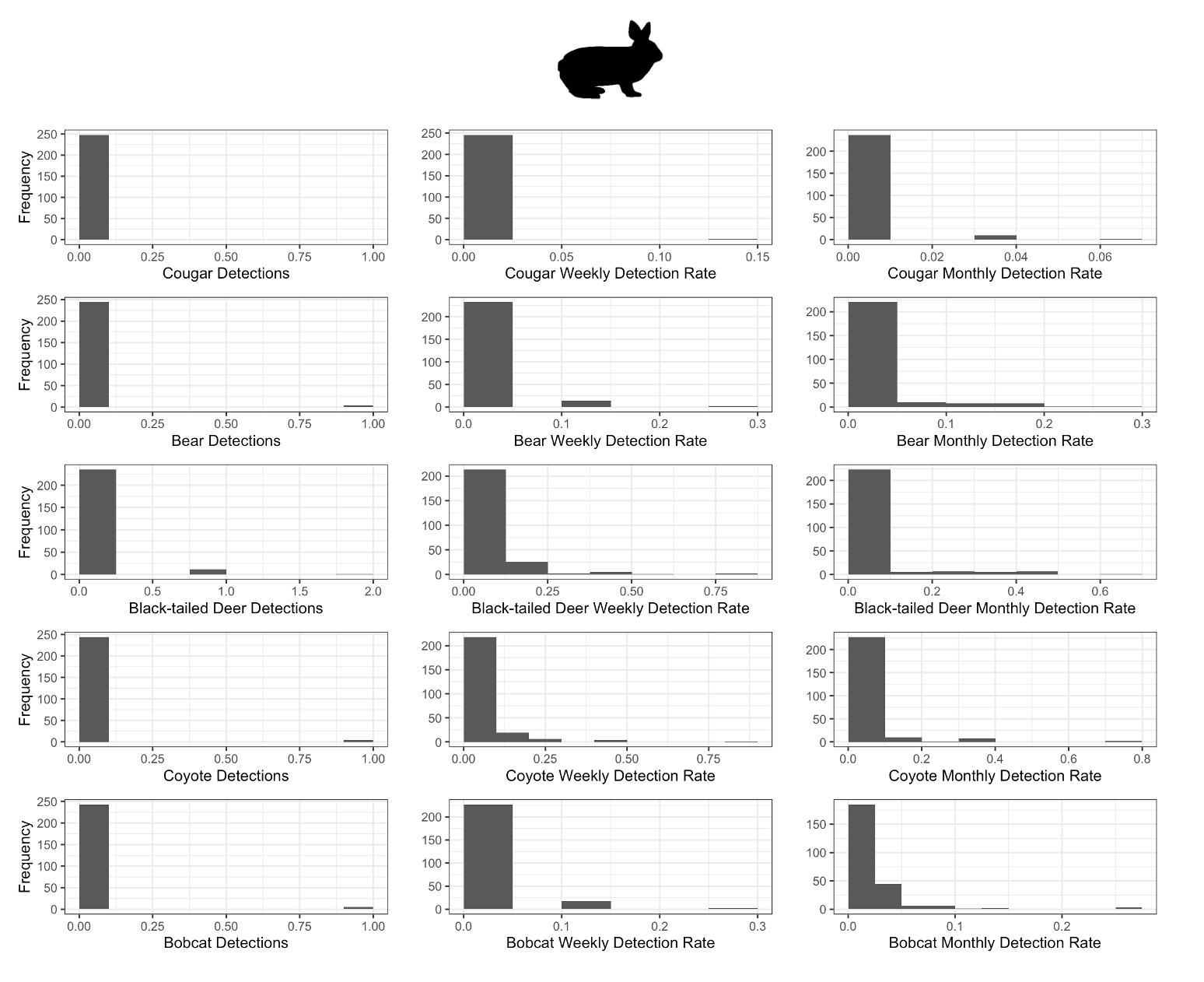

Supplement: S5 Fig — The snowshoe hare dataset comprises all independent detection events of snowshoe hares. Therefore, histograms show the detections of each species during that same day of the detection event (left column), the weekly detection rate during that same week of the detection event (middle column), and the monthly detection rate during that same month of the detection event (right column). Non-snowshoe hare species are cougars, black bears, black-tailed deer, coyotes, and bobcats (rows, from top to bottom). (PNG) [file pone.0286131.s005.png]

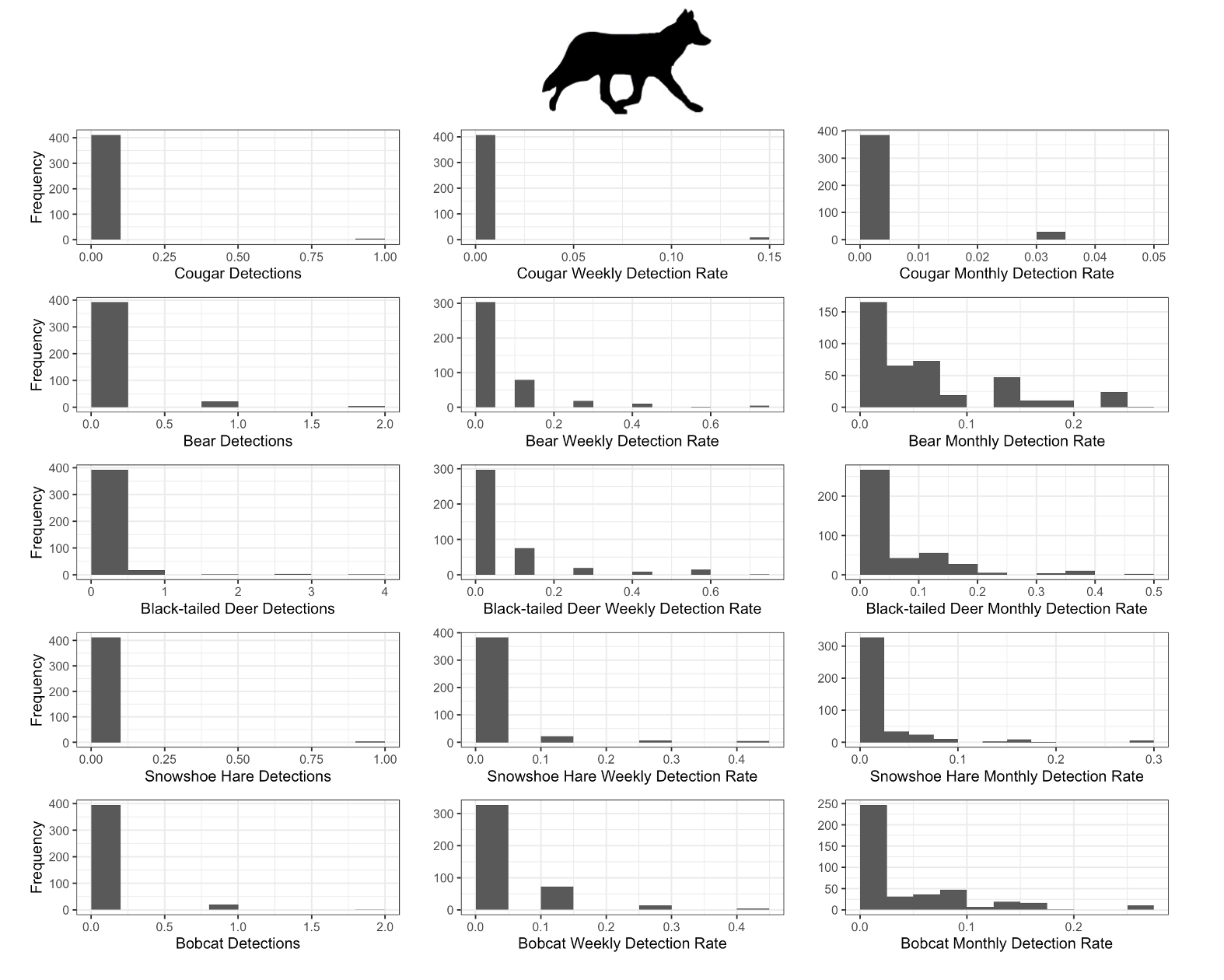

Supplement: S6 Fig — The coyote dataset comprises all independent detection events of coyotes. Therefore, histograms show the detections of each species during that same day of the detection event (left column), the weekly detection rate during that same week of the detection event (middle column), and the monthly detection rate during that same month of the detection event (right column). Non-coyote species are cougars, black bears, black-tailed deer, snowshoe hares, and bobcats (rows, from top to bottom). (PNG) [file pone.0286131.s006.png]

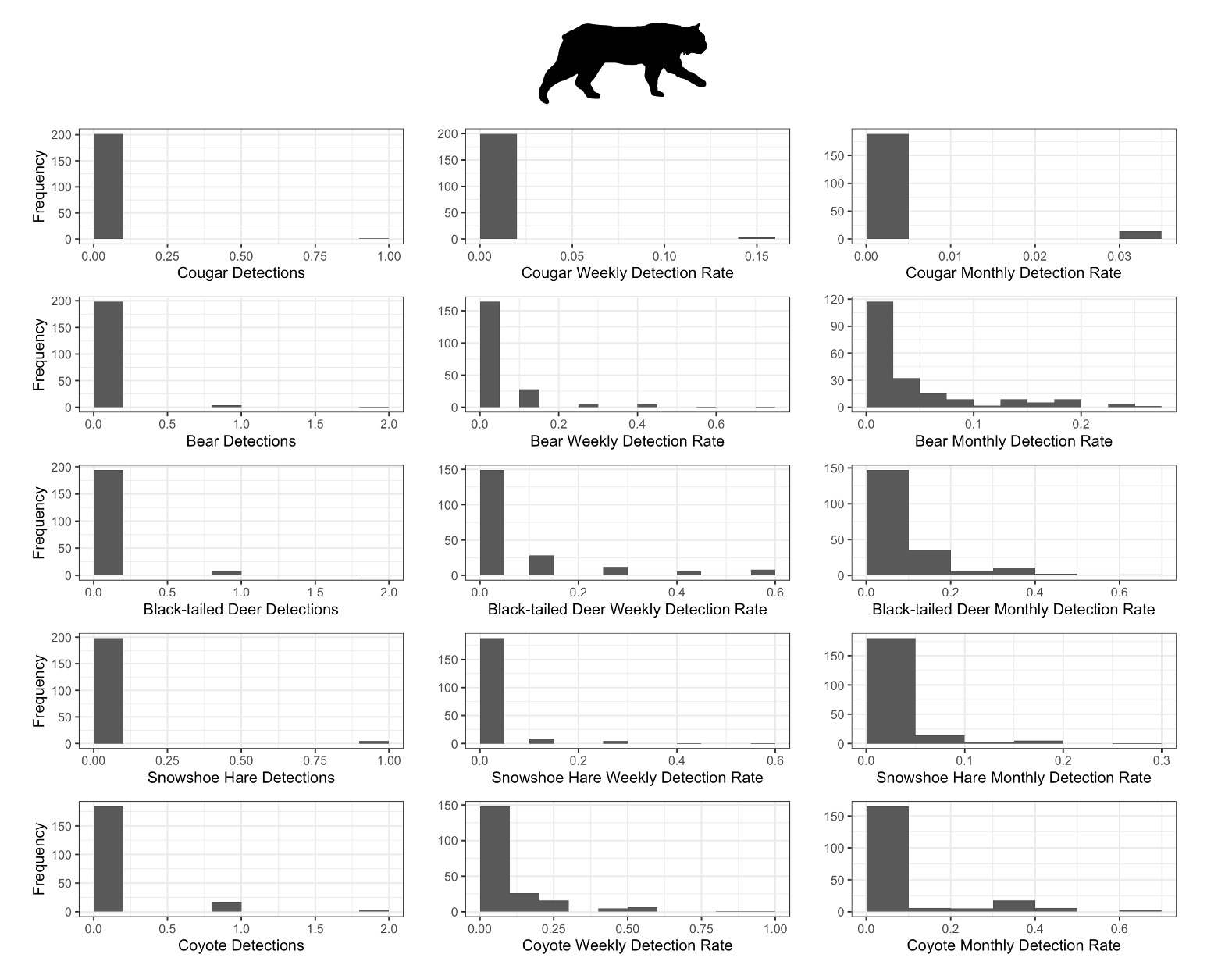

Supplement: S7 Fig — The bobcat dataset comprises all independent detection events of bobcats. Therefore, histograms show the detections of each species during that same day of the detection event (left column), the weekly detection rate during that same week of the detection event (middle column), and the monthly detection rate during that same month of the detection event (right column). Non-bobcat species are cougars, black bears, black-tailed deer, snowshoe hares, and coyotes (rows, from top to bottom). (PNG) [file pone.0286131.s007.png]

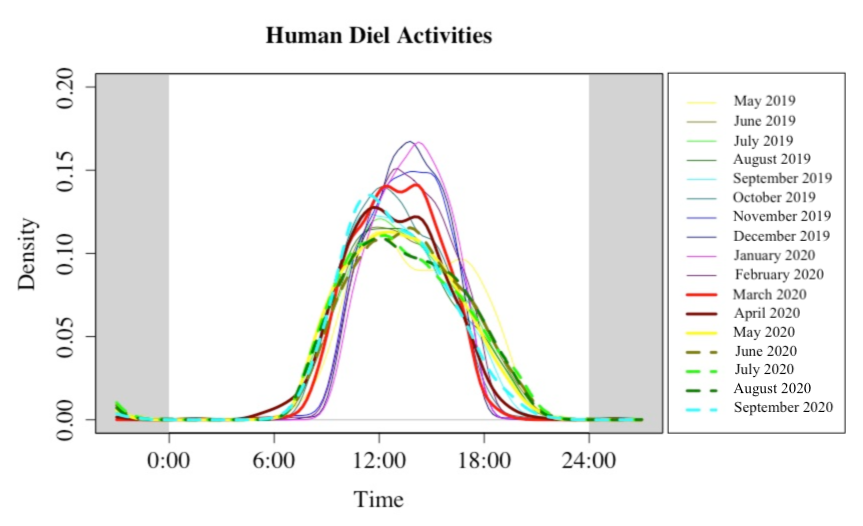

Supplement: S8 Fig — Each line represents the average kernel density (y-axis) of human detections throughout a 24-hour period (x-axis) for each month of camera trap sampling. (PNG) [file pone.0286131.s008.png]

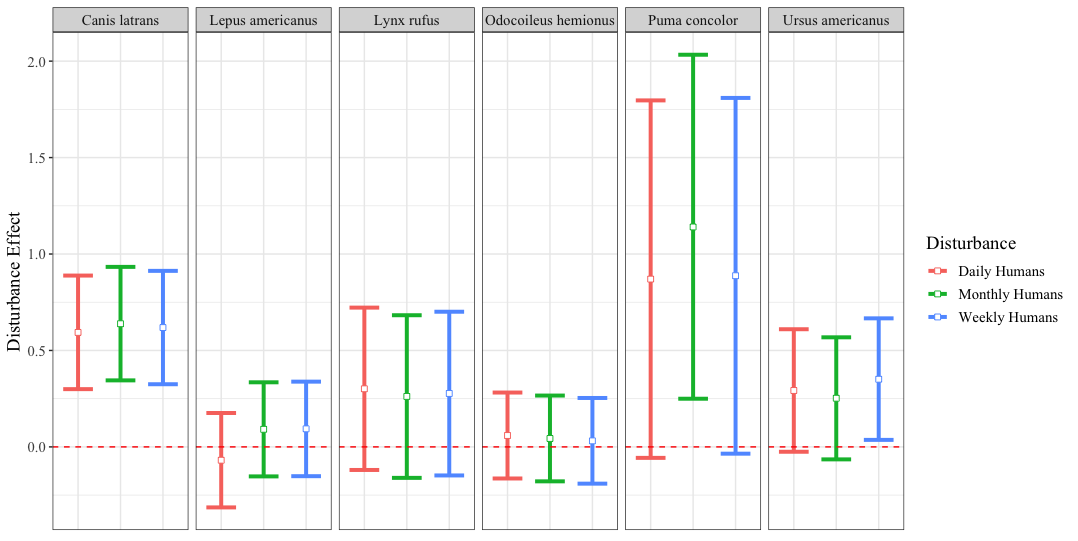

Supplement: S9 Fig — Each panel corresponds to a specific species, x-axes show different human-use variables included in univariate models, while y-axes show disturbance effect (parameter estimates). Center points in each line represent mean parameter estimates, while lines represent 95% credible intervals. (PNG) [file pone.0286131.s009.png]
