# Supplementary material for: Human presence and infrastructure impact wildlife nocturnality differently across an assemblage of mammalian species
Source: PLoS One. 2023 May 25;18(5):e0286131. doi: 10.1371/journal.pone.0286131 (PMC10212153; doi:10.1371/journal.pone.0286131)
Supplement: S1 Table — All species’ nocturnality data showed no signal of spatial autocorrelation, with the exception of coyotes (p < 0.05). (DOCX) [file pone.0286131.s010.docx]

**Table S1: Results of testing for spatial autocorrelation with Moran’s I tests.**

All species’ nocturnality data showed no signal of spatial autocorrelation, with the exception of coyotes (p < 0.05). * Denotes significant results.

| Species | Moran’s I | p-value |
| --- | --- | --- |
| *Puma concolor* | 0.18 | 0.13 |
| *Ursus americanus* | -0.07 | 0.62 |
| *Odocoileus hemionus* | -0.03 | 0.49 |
| *Lepus americanus* | -0.02 | 0.44 |
| *Canis latrans* | 0.25 | 0.04* |
| *Lynx rufus* | 0.18 | 0.12 |
