# Supplementary material for: Human presence and infrastructure impact wildlife nocturnality differently across an assemblage of mammalian species
Source: PLoS One. 2023 May 25;18(5):e0286131. doi: 10.1371/journal.pone.0286131 (PMC10212153; doi:10.1371/journal.pone.0286131)
Supplement: S2 Table — These models contrasted wildlife nocturnality against 1) the number of humans detected during the day of the wildlife detection event (i.e., “daily human detections”), 2) the number of humans detected throughout the week of the wildlife detection event divided by the number of days the camera was active (i.e., “weekly human detection rate”), and 3) the number of humans detected throughout the month of the wildlife detection event (i.e., “monthly human detection rate”). For each species, the model with the greatest Bayes Factor (bolded) was assumed to best explain variation in the data and was therefore used in construction of subsequent models. If no model outperformed the intercept-only model for a given species (all model Bayes Factors < 1), the species was modeled without a measure of direct human activity. (DOCX) [file pone.0286131.s011.docx]

**Table S1: Bayes Factors associated with the three preliminary models constructed for each species.**

These models contrasted wildlife nocturnality against 1) the number of humans detected during the day of the wildlife detection event, 2) the average detection rate of humans detected per day throughout the week of the wildlife detection event, and 3) the average detection rate of humans detected per day throughout the month of the wildlife detection event. For each species, the model with the greatest Bayes Factor (bolded) was assumed to best explain variation in the data and was therefore used in construction of subsequent models. If no model outperformed the intercept-only model for a given species (all model Bayes Factors < 1), the species was modeled without a measure of direct human activity.

| Species | Model | Bayes Factor |
| --- | --- | --- |
| *Puma concolor* | nocturnality ~ daily humans | 6.76 |
|  | nocturnality ~ weekly humans | 7.35 |
|  | **nocturnality ~ monthly humans** | **25.69** |
|  | nocturnality ~ 1 (intercept-only) | 1.00 |
| *Ursus americanus* | nocturnality ~ daily humans | 2.11 |
|  | **nocturnality ~ weekly humans** | **4.30** |
|  | nocturnality ~ monthly humans | 1.38 |
|  | nocturnality ~ 1 (intercept-only) | 1.00 |
| *Odocoileus hemionus* | nocturnality ~ daily humans | 0.33 |
|  | nocturnality ~ weekly humans | 0.29 |
|  | nocturnality ~ monthly humans | 0.31 |
|  | nocturnality ~ 1 (intercept-only) | 1.00 |
| *Lepus americanus* | nocturnality ~ daily humans | 0.36 |
|  | nocturnality ~ weekly humans | 0.41 |
|  | nocturnality ~ monthly humans | 0.41 |
|  | nocturnality ~ 1 (intercept-only) | 1.00 |
| *Canis latrans* | nocturnality ~ daily humans | 845.90 |
|  | nocturnality ~ weekly humans | 1651.21 |
|  | **nocturnality ~ monthly humans** | **2937.28** |
|  | nocturnality ~ 1 (intercept-only) | 1.00 |
| *Lynx rufus* | **nocturnality ~ daily humans** | **1.45** |
|  | nocturnality ~ weekly humans | 1.24 |
|  | nocturnality ~ monthly humans | 1.14 |
|  | nocturnality ~ 1 (intercept-only) | 1.00 |
