# Supplementary material for: Human presence and infrastructure impact wildlife nocturnality differently across an assemblage of mammalian species
Source: PLoS One. 2023 May 25;18(5):e0286131. doi: 10.1371/journal.pone.0286131 (PMC10212153; doi:10.1371/journal.pone.0286131)
Supplement: S3 Table — a “Dist. to bound” = Distance to the urban-wildland boundary (m). (DOCX) [file pone.0286131.s012.docx]

Table S2: Parameter estimates, 95% credible intervals, and 90% credible intervals from Bayesian regression models which contrasted wildlife nocturnality against measures of human activity, and landscape measures of implied human activity, while controlling for alternative factors (e.g., environmental variables) which might also influence wildlife diel activities.

*^a^* “Dist. to bound” = Distance to the urban-wildland boundary (m).

| Species | Predictors | Estimate | 95% L | 95% U | 90% L | 90% U |
| --- | --- | --- | --- | --- | --- | --- |
| *Puma concolor* | Intercept | 6.48 | 5.59 | 7.36 | 5.74 | 7.22 |
|  | Monthly human detection rate | 0.70 | -0.38 | 1.78 | -0.20 | 1.60 |
|  | **Road density** | -1.14 | -2.36 | 0.09 | **-2.16** | **-0.12** |
|  | Trail density | 0.54 | -0.48 | 1.56 | -0.31 | 1.39 |
|  | Dist. to Bound. *^a^* | -0.71 | -1.94 | 0.51 | -1.73 | 0.31 |
|  | Lunar phase | -0.10 | -1.02 | 0.81 | -0.87 | 0.66 |
|  | Crown closure | -0.30 | -1.23 | 0.63 | -1.08 | 0.47 |
| *Ursus americanus* | Intercept | 4.70 | 4.39 | 5.01 | 4.44 | 4.96 |
|  | **Weekly human detection rate** | 0.35 | -0.01 | 0.71 | **0.04** | **0.65** |
|  | Road density | 0.09 | -0.43 | 0.61 | -0.35 | 0.52 |
|  | Trail density | 0.04 | -0.35 | 0.43 | -0.28 | 0.37 |
|  | Dist. to Bound. | -0.10 | -0.52 | 0.32 | -0.46 | 0.25 |
|  | Lunar phase | -0.03 | -0.35 | 0.28 | -0.30 | 0.23 |
|  | **Crown closure** | -0.35 | **-0.68** | **-0.02** | -0.63 | -0.07 |
| *Odocoileus hemionus* | Intercept | 5.74 | 5.52 | 5.96 | 5.55 | 5.92 |
|  | Road density | -0.13 | -0.44 | 0.18 | -0.39 | 0.13 |
|  | Trail density | -0.17 | -0.46 | 0.11 | -0.42 | 0.07 |
|  | **Dist. to Bound.** | -0.25 | -0.49 | 0.00 | **-0.45** | **-0.04** |
|  | Lunar phase | 0.06 | -0.16 | 0.28 | -0.13 | 0.25 |
|  | Crown closure | -0.02 | -0.25 | 0.21 | -0.21 | 0.17 |
| *Lepus americanus* | Intercept | 9.23 | 8.99 | 9.46 | 9.03 | 9.43 |
|  | Road density | -0.22 | -0.50 | 0.05 | -0.45 | 0.01 |
|  | **Trail density** | -0.53 | **-0.77** | **-0.29** | -0.73 | -0.33 |
|  | **Dist. to Bound.** | -0.24 | -0.50 | 0.03 | **-0.46** | **-0.01** |
|  | Lunar phase | -0.09 | -0.33 | 0.15 | -0.29 | 0.11 |
|  | Crown closure | 0.16 | -0.08 | 0.40 | -0.04 | 0.36 |
| *Canis latrans* | Intercept | 7.27 | 6.99 | 7.55 | 7.03 | 7.51 |
|  | Monthly human detection rate | 0.28 | -0.07 | 0.64 | -0.02 | 0.58 |
|  | **Road density** | -0.87 | **-1.29** | **-0.46** | -1.22 | -0.52 |
|  | **Trail density** | 0.81 | **0.46** | **1.17** | 0.51 | 1.11 |
|  | **Dist. to Bound.** | -0.29 | -0.59 | 0.01 | **-0.55** | **-0.04** |
|  | **Lunar phase** | 0.27 | -0.01 | 0.56 | **0.03** | **0.51** |
|  | **Crown closure** | 0.27 | -0.04 | 0.58 | **0.01** | **0.53** |
| *Lynx rufus* | Intercept | 8.11 | 7.69 | 8.54 | 7.76 | 8.47 |
|  | Daily human detections | 0.25 | -0.22 | 0.72 | -0.15 | 0.65 |
|  | Road density | -0.09 | -0.65 | 0.49 | -0.56 | 0.39 |
|  | Trail density | -0.07 | -0.57 | 0.44 | -0.49 | 0.36 |
|  | Dist. to Bound. | 0.01 | -0.47 | 0.48 | -0.39 | 0.41 |
|  | Lunar phase | -0.17 | -0.60 | 0.26 | -0.53 | 0.19 |
|  | Crown closure | -0.28 | -0.73 | 0.17 | -0.66 | 0.09 |
