# Supplementary material for: Human presence and infrastructure impact wildlife nocturnality differently across an assemblage of mammalian species
Source: PLoS One. 2023 May 25;18(5):e0286131. doi: 10.1371/journal.pone.0286131 (PMC10212153; doi:10.1371/journal.pone.0286131)
Supplement: Attachment — Submitted filename: Reviewer_Human_presence_and_infrastructure_PLOS.pdf [file pone.0286131.s013.pdf]

Review of “Human disturbance and infrastructure impact wildlife nocturnality differentially across an assemblage of mammalian species”

The manuscript describes a research project focused on assessing the effect of human active potential disturbance and infrastructure presence on a set of mammalian species along a gradient of human footprint in western Canada.

I found the manuscript interesting and well written. I list my comments and suggestions below:

Introduction and Discussion are well written and interesting.

Line 46: Instead of saying that ‘PAs are often thought to provide refuge..’ I would rather say that PAs <u>should</u> provide refuge from anthropogenic pressures, since we established PAs for that very reason, and if they do not offer any refuge from anthropogenic pressures then they are failing to fulfil their scope. I suggest you should deepen this point a little bit in this part of the Intro.

Lines 64-67: Perhaps I misunderstood your point here, but what do you mean exactly when you say that different species may respond to human disturbance at different temporal scales? Why would fine-scale changes in human presence not affect species that avoid seasonal or monthly patterns in human disturbance? Is there any evidence of animals responding to e.g. increase in tourism in summer not responding to daily changes in human activity?

Line 73: you already used the verb underscore at line 56.

Lines 76-77: Is this ability really linked positively to body-mass? I would say is rather typical of generalist species, that can exploit disturbed areas by shifting to nocturnality, while more sensitive species usually avoid disturbed areas also spatially, or spatio-temporally and not only temporally.

Line 92: Isn’t wildlife an uncountable noun? I think ‘wildlife <u>is</u> active’ is the correct form.

Line 95: Concerning human disturbance with social media data I think this research could be very useful here: Corradini, A., Randles, M., Pedrotti, L., van Loon, E., Passoni, G., Oberosler, V., ... & Cagnacci, F. (2021). Effects of cumulated outdoor activity on wildlife habitat use. Biological Conservation, 253, 108818. https://doi.org/10.1016/j.biocon.2020.108818

Line 112: I find the term ‘human-related infrastructure’ odd, isn’t infrastructure intrinsically human related?

Lines 141-147: When describing the study area you do not mention that British Columbia is part of Canada, nor in which part of Canada it is located. Readers outside north America might not know where British Columbia is, nor what the acronym BC stands for.

Lines 153: I think you wanted to begin this sentence with ‘as a’ and not with ‘a’.

Line 163: I would erase the word ‘shaping’.

Lines 170-176: Here comes my main concern regarding your study: the sampling design is not based on a regular grid, thus cameras are set at very different distances one another. Sampling sites within Malcolm Knapp research forest are much closer compared to those within Golden Ears, and this may bias your results. Most probably sites in Malcolm Knapp will have a much higher level of spatial autocorrelation than those in Golden Ears. One way to overcome this would be to test if results of neighbouring sites are more

correlated than those of sites that are more far apart (as for example done here: Kolowski, J. M., Oley, J., & McShea, W. J. (2021). High-density camera trap grid reveals lack of consistency in detection and capture rates across space and time. *Ecosphere*, *12*(2), e03350. https://doi.org/10.1002/ecs2.3350).

Line 195: You now mention 'shifts' for the first time, and it is not very clear what you refer to here. More in general, I am not sure that shift is the right word to use for your variable, since it seems to indicate a deviation or change from a certain, known value, whereas you are indicating a temporal distance from noon, taken as a reference.

Lines 205-212: I think that here it is not well justified and explained why there would be a need to test human passage at these three temporal scales. I imagine that sites with high overall human passage also have high weekly and daily values. The only situation in which this does not hold would be if human frequentation was concentrated in a few days and scarce for the rest of the sampling period (is that the case for your study areas?), but this possibility can easily been tested beforehand. Also, if that was the case, could you please list how would mammalian species respond differently to disturbance occurring at these three temporal intervals, are there evidence and example from the literature?

Line 228: I think there should be a short explanation of what Bayes Factor factor is for readers that are not familiar with it.

Results section: Have you assessed if you get similar results using a different nocturnality index (as for example nocturnal/(diurnal+nocturnal) events ratio)? I wonder whether the choice of the response variable is affecting your analysis.

Line 285-287: This sentence is not well linked to what is written before and after. Why is this observation important, what does it entail?

Line 364: This likewise here confounds me a little, you jump from effects of urban areas to seasonality too abruptly.

Line 401: erase the 'a' from 'a urban spaces'.

Lines 413-415: Did you explicitly test whether roads and trails were more used in Golden Ears than Malcolm Knapp? It would be useful to report numerical output of such test here.
