# Supplementary material for: Human presence and infrastructure impact wildlife nocturnality differently across an assemblage of mammalian species
Source: PLoS One. 2023 May 25;18(5):e0286131. doi: 10.1371/journal.pone.0286131 (PMC10212153; doi:10.1371/journal.pone.0286131)
Supplement: Attachment — Submitted filename: Second round of review.pdf [file pone.0286131.s016.pdf]

Second round of review

Reviewer 2

Discussion, Lines 453-455: I think this is an interesting finding and I would maybe discuss it more broadly. Most of the species you targeted show on average a shift towards nocturnal behavior in presence of humans, even if BCI are not strictly 'significant' I still think it is an interesting finding.

Lines 479-481: Are these species really naturally nocturnal? Is there evidence from non-human-dominated landscapes that these species still maintain nocturnality? Even species classically considered strictly crepuscular or nocturnal have been reported to be more diurnal where human presence is low (see for example: Kamler, J. F., Jędrzejewska, B., & Jędrzejewski, W. (2007). Activity patterns of red deer in Białowieża National Park, Poland. *Journal of mammalogy*, *88*(2), 508-514.)

Line 587: I think this work is highly relevant here: Salvatori, M., Oberosler, V., Rinaldi, M., Franceschini, A., Truschi, S., Pedrini, P., & Rovero, F. (2023). Crowded mountains: Long-term effects of human outdoor recreation on a community of wild mammals monitored with systematic camera trapping. *Ambio*, 1-13. https://doi.org/10.1007/s13280-022-01825-w Indeed, increased nocturnality and spatial avoidance of humans might not directly translate into decreased fitness for mammals, at least in the temporal interval considered in this study (10 years). So spatio-temporal changes in behavior to decrease the probability to encounter humans could actually be effective strategies to cope with recreation. However I agree with you that some limitations to human accessibility in PAs are desirable, especially considering the increasing trends in outdoor recreation across the globe.
