# Supplementary material for: Human presence and infrastructure impact wildlife nocturnality differently across an assemblage of mammalian species
Source: PLoS One. 2023 May 25;18(5):e0286131. doi: 10.1371/journal.pone.0286131 (PMC10212153; doi:10.1371/journal.pone.0286131)
Supplement: Attachment — Submitted filename: Response to Reviewer Comments.docx [file pone.0286131.s015.docx]

**Response to Reviewer Comments**

Dear Dr. Rovero,

Thank you again for facilitating a timely review of our manuscript. We also thank the reviewers for their time spent reviewing our manuscript and the thoughtful feedback they provided. We have revised the manuscript according to the requested changes, and we hope that this improved manuscript is now suitable for publication in *PLOS ONE*. Below you will find our point-by-point responses to the reviewer’s recommendations (line numbers of reviewer comments refer to the original manuscript, but line numbers in our responses refer to the revised manuscript). We look forward to hearing from you and thank you again for your consideration.

**Reviewer #1**, general comments:

- Overall, the manuscript is well written, and I think it meets the rigorous standards for publication in the journal. The authors fairly addressed the requests I had on the first version of the manuscript, resolving the main criticism I moved to it. However, I still have a minor concern about the manuscript, and I think a some corrections are required.
  - We thank you for these comments. We have made revisions as requested, and hope that our manuscript now adequately addresses the issues raised.

**Reviewer #1**, line-by-line comments:

- L81: I appreciated your inclusion of the work from Wang et al., which explores another possible effect of human activity on wildlife; however, I would suggest to rephrase this part in something like:

  “increased temporal overlap between predator and prey species (causing greater predation risk (36)), and between competing predators (37), or effective habitat loss […]”

  since Wang et al. found a reduction in temporal segregation among coexisting predators, but they did not directly test whether this resulted in increased competition or not (as they clearly state in their discussion).
  - We have changed lines 81-82 to reflect this request:

    “...increased temporal overlap between predator and prey species (causing greater predation risk (36)), increased temporal overlap between competing predators (37), or effective habitat loss...”
- L216: Please provide the name and version of the software used to manually process the camera trap images.
  - We have added the name and version of the private software to lines 217-218:

    “...private cloud-based photo management software (WildCo CamTrap System v 3.0) (54).”
- L361 TABLE: I have to insist on this point. In the new version of the manuscript I still find a Table 1 at line 258, describing the “Predictor variables considered[…]”, which is correctly reported as Table 1 in the text. Then, on line 361 I find a second table, still named Table 1: “Summary of detections […]”, which is (correctly) reported within text as Table 2. Please clarify this.
  - We apologize for misunderstanding your original request. Line 363 now reads “Table 2”
- L345-348: This is the only (minor) concern I have about the manuscript. I think presenting the activity patterns of coyotes together with that of cougars and deer is a bit misleading. It is true that coyote activity has two “peaks” around dusk and down, but I would suggest caution in defining its activity as bimodal, as it never really drops during the night, conversely to what happens to cougar and especially deer activity. I would suggest to clarify that, also considering that based on Table 2 coyote is the third more nocturnal species (after hare and bobcat).
  - We have changed lines 346-354 to reflect this request:

    “Cougars and black-tailed deer maintained diel activity curves with mostly bimodal distributions, peaking at or near dawn and dusk (cougar activity peaked around 6:00 am and 9:00 pm, while black-tailed deer activity peaked at approximately 6:00 am and 6:00 pm; Fig. 2). Black bear activity was greatest from approximately 6:00 am to 9:00 pm, with activity steadily increasing from around 2:00 pm to 9:00 pm where it peaked and subsequently dropped (Fig. 2). Snowshoe hares, coyotes, and bobcats all showed nocturnal activity patterns, with snowshoe hare activity being greatest from approximately 10:00 pm to 4:00 am, coyote activity peaking around 6:00 am and 9:00 pm (and remaining active during nighttime hours), and bobcat activity peaking around 2:00 am (Fig. 2).”

**Reviewer #2,** general comments:

- I thank the Authors for carefully answering and considering all of my comments of the previous round. I think that after considering the few more comments I have given in this second round of review the manuscript will be ready to be published.
  - It does not appear that there were any additional comments from this reviewer. Nevertheless, we appreciate the reviewer taking the time to read our manuscript again, and hope that our latest revisions adequately address any remaining concerns.
