# Supplementary material for: Human presence and infrastructure impact wildlife nocturnality differently across an assemblage of mammalian species
Source: PLoS One. 2023 May 25;18(5):e0286131. doi: 10.1371/journal.pone.0286131 (PMC10212153; doi:10.1371/journal.pone.0286131)
Supplement: Attachment — Submitted filename: Response to Reviewer Comments.docx [file pone.0286131.s017.docx]

**Response to Reviewer Comments**

Dear Dr. Rovero,

Thank you again for facilitating a timely review of our manuscript. We also thank the reviewers for their time spent reviewing our manuscript and the thoughtful feedback they provided. We have revised the manuscript according to the requested changes, and we hope that this improved manuscript is now suitable for publication in *PLOS ONE*. Below you will find our point-by-point responses to the reviewer’s recommendations (line numbers of reviewer comments refer to the original manuscript, but line numbers in our responses refer to the revised manuscript). We look forward to hearing from you and thank you again for your consideration.

**Reviewer #1**, general comments:

- Overall, the manuscript is well written, and I think it meets the rigorous standards for publication in the journal. The authors fairly addressed the requests I had on the first version of the manuscript, resolving the main criticism I moved to it. However, I still have a minor concern about the manuscript, and I think a some corrections are required.
  - We thank you for these comments. We have made revisions as requested, and hope that our manuscript now adequately addresses the issues raised.

**Reviewer #1**, line-by-line comments:

- L81: I appreciated your inclusion of the work from Wang et al., which explores another possible effect of human activity on wildlife; however, I would suggest to rephrase this part in something like:

  “increased temporal overlap between predator and prey species (causing greater predation risk (36)), and between competing predators (37), or effective habitat loss […]”

  since Wang et al. found a reduction in temporal segregation among coexisting predators, but they did not directly test whether this resulted in increased competition or not (as they clearly state in their discussion).
  - We have changed lines 81-82 to reflect this request:

    “...increased temporal overlap between predator and prey species (causing greater predation risk (36)), increased temporal overlap between competing predators (37), or effective habitat loss...”
- L216: Please provide the name and version of the software used to manually process the camera trap images.
  - We have added the name and version of the private software to lines 217-218:

    “...private cloud-based photo management software (WildCo CamTrap System v 3.0) (54).”
- L361 TABLE: I have to insist on this point. In the new version of the manuscript I still find a Table 1 at line 258, describing the “Predictor variables considered[…]”, which is correctly reported as Table 1 in the text. Then, on line 361 I find a second table, still named Table 1: “Summary of detections […]”, which is (correctly) reported within text as Table 2. Please clarify this.
  - We apologize—we misunderstood your original request. Line 363 now reads “Table 2”
- L345-348: This is the only (minor) concern I have about the manuscript. I think presenting the activity patterns of coyotes together with that of cougars and deer is a bit misleading. It is true that coyote activity has two “peaks” around dusk and down, but I would suggest caution in defining its activity as bimodal, as it never really drops during the night, conversely to what happens to cougar and especially deer activity. I would suggest to clarify that, also considering that based on Table 2 coyote is the third more nocturnal species (after hare and bobcat).
  - We have changed lines 346-354 to reflect this request:

    “Cougars and black-tailed deer maintained diel activity curves with mostly bimodal distributions, peaking at or near dawn and dusk (cougar activity peaked around 6:00 am and 9:00 pm, while black-tailed deer activity peaked at approximately 6:00 am and 6:00 pm; Fig. 2). Black bear activity was greatest from approximately 6:00 am to 9:00 pm, with activity steadily increasing from around 2:00 pm to 9:00 pm where it peaked and subsequently dropped (Fig. 2). Snowshoe hares, coyotes, and bobcats all showed nocturnal activity patterns, with snowshoe hare activity being greatest from approximately 10:00 pm to 4:00 am, coyote activity peaking around 6:00 am and 9:00 pm (and remaining active during nighttime hours), and bobcat activity peaking around 2:00 am (Fig. 2).”

**Reviewer #2, line-by-line comments:**

- Discussion, Lines 453-455: I think this is an interesting finding and I would maybe discuss it more broadly. Most of the species you targeted show on average a shift towards nocturnal behavior in presence of humans, even if BCI are not strictly ‘significant’ I still think it is an interesting finding.
  - We agree that this is an interesting finding that deserves more discussion beyond what is briefly stated in lines 453-455. However, we already addressed this in other areas of the manuscript. In lines 507-530, we further discuss the trends of all carnivores being predominantly more nocturnal, despite a lack of “significance”. Specifically, in lines 510-516, we discuss how human activity is predictably diurnal in this study area, which may allow carnivores to adequately segregate by becoming only slightly more nocturnal (e.g., becoming more crepuscular). We then mention how small shifts towards increased crepuscular activity might lead to smaller effect sizes in lines 516-518, which could help to explain the lack of “significance”. Furthermore, in lines 524-530 we discuss how individual behavioral variation may have produced greater uncertainty (wider CIs) in our estimates and made it more difficult to make broader claims about the overall “population-level” impacts on these diverse individuals. We aimed to keep these speculative discussion points to a minimum, but we hope that the ideas raised in this section adequately discuss the broader implications of this finding.
- Lines 479-481: Are these species really naturally nocturnal? Is there evidence from non-human-dominated landscapes that these species still maintain nocturnality? Even species classically considered strictly crepuscular or nocturnal have been reported to be more diurnal where human presence is low (see for example: Kamler, J. F., Jędrzejewska, B., & Jędrzejewski, W. (2007). Activity patterns of red deer in Białowieża National Park, Poland. *Journal of mammalogy*, *88*(2), 508-514.)
  - This is a very interesting point—thank you for bringing this up. To address this, we have added lines 455-460:
    “...additional research would be needed to confirm whether these species are indeed nocturnal in areas without human presence. Some species which are typically thought of as nocturnal or crepuscular may exhibit diurnal behavior in areas where humans or predators are excluded (51,78), so there is also a need for additional monitoring in areas without humans to understand the full range of wildlife diel activities both with and without human influences.”
- Line 587: I think this work is highly relevant here: Salvatori, M., Oberosler, V., Rinaldi, M., Franceschini, A., Truschi, S., Pedrini, P., & Rovero, F. (2023). Crowded mountains: Long-term effects of human outdoor recreation on a community of wild mammals monitored with systematic camera trapping. *Ambio*, 1-13. https://doi.org/10.1007/s13280-022-01825-w Indeed, increased nocturnality and spatial avoidance of humans might not directly translate into decreased fitness for mammals, at least in the temporal interval considered in this study (10 years). So spatio-temporal changes in behavior to decrease the probability to encounter humans could actually be effective strategies to cope with recreation. However I agree with you that some limitations to human accessibility in PAs are desirable, especially considering the increasing trends in outdoor recreation across the globe.
  - We thank you for including this new paper. We have incorporated it into our final discussion point at lines 570-572:
    “... long-term camera trapping studies have also shown increasing occupancy of high human use areas by of a number of wildlife species when they are able to adequately segregate via increased nocturnality (88).”
